# Stress cardiac magnetic resonance imaging in an outpatient setting

**DOI:** 10.1186/1532-429X-11-S1-P85

**Published:** 2009-01-28

**Authors:** Amir-Ali Fassa, Alain Naïmi, Golmehr Ashrafpoor, Yannick Magliano, Béatrice Veragut Davies, Juan Sztajzel

**Affiliations:** 1 Geneva University Hospitals, Geneva, Switzerland; 2 Centre de Diagnostic Radiologique de Carouge, Geneva, Switzerland; 3 Centre Cardio-Pulmonaire de la Clinique de Carouge, Geneva, Switzerland

**Keywords:** Cardiac Magnetic Resonance, Thallium, Dobutamine, Cardiac Magnetic Resonance Imaging, Myocardial Scar

## Introduction

Stress cardiac magnetic resonance (CMR) imaging is a non-invasive modality which is increasingly used for detection of myocardial ischemia, necrosis and viability.

## Purpose

To assess the feasibility and safety of stress CMR in a non-hospital outpatient setting.

## Methods

We reviewed the data of all patients who were referred for stress CMR (1.5 Tesla) from February 1^st^ 2006 to January 31^st^ 2008 to the Centre de Diagnostic Radiologique de Carouge, an outpatient imaging centre. Standard protocol consisted of: 1) assessment of myocardial function at rest; 2) pharmacological stress induced either by dobutamine (protocol of 10, 20, 30, 40 μg/kg/min during 3 minutes with atropine if necessary) until achieving submaximal heart rate ([220-age] × 0.85), or by adenosine (protocol of 140 μg/kg/minute during 3 minutes followed by a bolus of 10 ml of gadolinium at 4 ml/second "first pass"); 3) assessment of myocardial scar and/or viability by delayed enhancement (DE) sequences.

## Results

During the study period 472 patients were referred for stress CMR. The test was performed in 452 patients (96%): 294 males (65%), mean age 62 ± 11 years, mean duration 55 ± 10 minutes, stress induction with dobutamine in 241 patients (53%) and adenosine in 211 patients (47%). The test could not be carried out in 23 patients (5%) because of claustrophobia (18 patients), excessive thoracic diameter (4 patients) and excessive baseline arterial blood pressure (1 patient). However, stress CMR could finally be performed during a second appointment in 3 patients. The remainder either underwent another non-invasive test (Thallium myocardial scintigraphy in 14 patients), or did not undergo further functional assessment at our centre (6 patients). No ischemia or infarction was found in 306 patients (68%), while isolated ischemia was found in 18 patients (4%) and ischemia in the presence of an infarction in 30 patients (7%). Infarction without ischemia was found in 95 patients (21%). DE was found 146 patients (33%), which was subendocardial in 124 patients (28%, involving <50% of wall thickness in 53 patients (12%) and >50% of wall thickness in 71 patients (16%)). DE sparing the subendocardium was found in 22 patients (5%). The following complications occurred during 17 tests (4%): supraventricular tachycardia or unsustained ventricular tachycardia (7 patients), anxiety attack (3 patients), chest pain (3 patients), suspected allergic reaction to gadolinium (1 patient), dizziness (1 patient), vomiting during dobutamine infusion (1 patient) and hypotension (1 patient). No other complications occurred.

## Conclusion

Stress CMR is a well tolerated non-invasive assessment modality, which can be safely performed in a non-hospital outpatient setting.

